# Insulin Mimetic Properties of Extracts Prepared from *Bellis perennis*

**DOI:** 10.3390/molecules23102605

**Published:** 2018-10-11

**Authors:** Renate Haselgrübler, Verena Stadlbauer, Flora Stübl, Bettina Schwarzinger, Ieva Rudzionyte, Markus Himmelsbach, Marcus Iken, Julian Weghuber

**Affiliations:** 1 School of Engineering, University of Applied Sciences Upper Austria, Stelzhamerstrasse 23, A-4600 Wels, Austria; renate.haselgruebler@fh-wels.at (R.H.); flora.stuebl@fh-wels.at (F.S.); bettina.schwarzinger@fh-wels.at (B.S.); ieva.rudzionyte@fh-wels.at (I.R.); 2 Austrian Competence Center for Feed and Food Quality, Safety and Innovation, A-4600 Wels, Austria; 3 Institute for Analytical Chemistry, Johannes Kepler University, A-4040 Linz, Austria; markus.himmelsbach@jku.at; 4 PM International AG, L-5445 Schengen, Luxembourg; marcus.iken@pm-international.de

**Keywords:** *Bellis perennis* extract, insulin mimetic property, GLUT4 translocation, glucose uptake, hens egg test-chorioallantoic membrane (HET-CAM) assay, type 2 diabetes

## Abstract

Diabetes mellitus (DM) and consequential cardiovascular diseases lead to millions of deaths worldwide each year; 90% of all people suffering from DM are classified as Type 2 DM (T2DM) patients. T2DM is linked to insulin resistance and a loss of insulin sensitivity. It leads to a reduced uptake of glucose mediated by glucose transporter 4 (GLUT4) in muscle and adipose tissue, and finally hyperglycemia. Using a fluorescence microscopy-based screening assay we searched for herbal extracts that induce GLUT4 translocation in the absence of insulin, and confirmed their activity in chick embryos. We found that extracts prepared from *Bellis perennis* (common daisy) are efficient inducers of GLUT4 translocation in the applied in vitro cell system. In addition, these extracts also led to reduced blood glucose levels in chicken embryos (in ovo), confirming their activity in a living organism. Using high-performance liquid chromtaography (HPLC) analysis, we identified and quantified numerous polyphenolic compounds including apigenin glycosides, quercitrin and chlorogenic acid, which potentially contribute to the induction of GLUT4 translocation. In conclusion, *Bellis perennis* extracts reduce blood glucose levels and are therefore suitable candidates for application in food supplements for the prevention and accompanying therapy of T2DM.

## 1. Introduction

Diabetes mellitus is a group of metabolic diseases leading to hyperglycemia. People with type 2 diabetes mellitus (T2DM) represent the main group of patients with diabetes (90%) around the world [1]. The global prevalence of diabetes has increased significantly within the last decades with more than 400 million affected people in 2014. Diabetes caused 1.5 million deaths in 2012 and an additional 2.2 million deaths by increasing the risks of cardiovascular and other diseases [2]. Apart from health issues, diabetes threatens the economies of all nations. The absolute global costs of diabetes and its consequences are large and will substantially increase by 2030: the increase in costs as a share of the global gross domestic product has been reported to grow from 1.8% in 2015 to a maximum of 2.2% by 2030 [3].

The main features of T2DM are hyperglycemia, insulin resistance and obesity. Additionally, T2DM is also associated with hyperlipidemia and hypertension. This combination is known as the metabolic syndrome and is a high-risk factor for cardiovascular diseases [4,5]. For this reason, there is a great demand for approaches to prevent and treat the health issues associated with T2DM. Glucose-lowering pharmacotherapy includes insulin sensitizers such as glitazone [6], inhibitors of hepatic gluconeogenesis such as metformin [7], or renal sodium-glucose co-transporter (SGLT1/2) inhibitors such as dapagliflozin [8]. However, application of these drugs is associated with severe side effects including diarrhea, nausea, abdominal pain, edema, congestive heart failure, glycosuria followed by urinary tract infections, and possibly bladder cancer [9,10,11,12,13].

Many plants that are consumed as nutraceuticals or in traditional Chinese medicine contain active ingredients, termed phytochemicals, which have potential anti-diabetic properties. These effects are based on the modulation of various cellular and physiological pathways, such as the increase in insulin sensitivity, the inhibition of lipid absorption and metabolism, or an increase of glucose uptake in muscle and adipose tissue [14]. Moreover, the inhibition of intestinal glucose absorption, which is predominantly facilitated by sodium-dependent glucose co-transporter 1 (SGLT1) and glucose transporter 2 (GLUT2), represents an attractive alternative for the prevention of high blood glucose levels. For example, we have recently shown that extracts prepared from Guava (*Psidium guajava*) effectively inhibit intestinal glucose absorption [15].

As reported previously, our lab is aiming to identify and characterize phytochemicals, termed insulin mimetics, which induce the uptake of glucose into muscle and adipose tissue in the absence of insulin [16,17]. These compounds potentially induce the translocation of glucose transporter 4 (GLUT4) from cytosolic compartments into the plasma membrane [18,19], which ultimately leads to decreased blood glucose levels. The underlying mechanisms are not fully understood but, for example, it has been shown that some polyphenols activate AMP-activated protein kinase (AMPK) [20], or phosphatidylinositide 3 (PI3) kinase [21], whose activation results in an increased GLUT4 translocation.

For a screening approach, we apply a highly sensitive fluorescence microscopy-based assay to quantify the GLUT4 translocation process [16]. We also use wet lab chemistry approaches to verify the effects of putative positive hits, and, more importantly, test their efficacy in vivo. For this purpose, a modified hens egg test (termed Gluc-HET; [22,23]), which was developed in our lab on the basis of the well-established hens egg test-chorioallantoic membrane (HET-CAM) assay [24], has turned out to be a promising strategy. Importantly, experiments performed with non-hatched avian embryos in the first two-thirds of embryonic development (lasting 21 days) are not considered animal experiments. Therefore, approval by an ethics committee is not required. Serum insulin levels are not detectable until day 12 of development, and therefore interference between naturally produced insulin and insulin mimetic compounds applied until day 12 can be regarded as non-relevant [25]. Taken together, the Gluc-HET system represents a valuable tool to test insulin mimetic compounds in a living organism.

Here, we describe the identification and characterization of extracts prepared from *Bellis perennis* (common daisy) as promising compound mixtures that effectively reduce blood glucose levels in ovo, most likely by the induction of GLUT4 translocation, which leads to increased glucose uptake from the blood circuit.

## 2. Materials and Methods

### 2.1. Reagents

Human insulin, CaCl_2_, NaCl, KCl, MgSO_4_, KH_2_PO_4_, phosphate buffered saline (PBS), Hank’s balanced salt solution (HBSS), quercetin, chlorogenic acid, neochlorogenic acid and caffeic acid were purchased from Sigma-Aldrich (Schnelldorf, Germany). Guaijaverin and avicularin were from Glentham Life Sciences (Corsham, UK). Rutin, hyperoside, isoquercitrin and quercitrin were obtained from Extrasynthese (Genay CEDEX, France). For preparation of stock solutions, the herbal compounds were dissolved in Krebs Ringer phosphate HEPES buffer (KRPH; 20 mM HEPES, 1 mM CaCl_2_, 136 mM NaCl, 4.7 mM KCl, 1 mM MgSO_4_ and 5 mM KH_2_PO_4_). A library containing 2300 water-soluble herbal extracts (PECKISH) [26] was provided by Frank Döring (Christian-Albrechts University, Kiel, Germany). NovoRapid manufactured by Novo Nordisk was a kind gift from Daniel Weghuber (Paracelsus Medical University, Salzburg, Austria). A saponin mix with 10% saponin content was provided by Delacon Biotechnik GmbH (Steyregg, Austria). Transwell inserts (8.4 mm, collagen-treated, 0.4 μm pore diameter) and 24-well plates were obtained from Greiner Bio-One (Kremsmünster, Austria).

### 2.2. Cell Culture and Transfection

CHO-K1 cells stably expressing human insulin receptor (hIR) and GLUT4-myc-GFP were a kind gift from Manoj K. Bhat (National Centre for Cell Science, University of Pune, India). Cells were maintained in Ham’s F12 culture medium supplemented with 100 μg/mL penicillin, 100 μg/mL streptomycin, 1% G418 and 10% fetal bovine serum (FBS) (all Life Technologies, Carlsbad, CA, USA). Human Caco-2 cells were obtained from DSMZ (Braunschweig, Germany) and maintained in MEM with Earle’s salts supplemented with 10% FBS, 100 μg/mL penicillin, 100 µg/mL streptomycin, and 0.1% 2-mercaptoethanol (all Life Technologies, Carlsbad, CA, USA). The cells were grown at 37 °C in a humidified atmosphere with 5% CO_2_.

### 2.3. Cytotoxicity Assay

Cytotoxic effects of compounds used for the cell layer integrity study were evaluated by using a resazurin-based in vitro toxicology assay (Sigma-Aldrich; Schnelldorf, Germany), according to the manufacturer’s instructions. Briefly, cells were seeded into 96-well plates (45,000 cells per well), grown to 90% confluence, and incubated with the test substances for 2 h at 37 °C. The cells were washed and incubated with 10% resazurin in growth medium for 2 h. Subsequently, the amount of the reduced form of resazurin (resorufin) was determined with a microplate reader in fluorescence mode (544 nm excitation, 590 nm emission; POLARstar Omega, BMG LABTECH, Ortenberg, Germany). Data analysis was done by OmegaMARS Data analysis software package (BMG LABTECH, Ortenberg, Germany). Untreated cells grown under the same conditions were used for normalization of cell viability. Each test substance was measured in triplicate.

### 2.4. Determination of Cell Layer Integrity by Transepithelial Electrical Resistance (TEER) Measurements and Sugar Transport Quantitation

For cell layer integrity and sugar transport measurements, Caco-2 cells were seeded at 1.65 × 10^5^ cells/insert. Differentiation was induced the following day with Entero-STIM Intestinal Epithelium Differentiation Medium (Corning, Wiesbaden, Germany) supplemented with 100 μg/mL penicillin, 100 µg/mL streptomycin and 0.1% MITO + Serum Extender (Corning, Wiesbaden, Germany). Daily change of cell medium was followed by the experiment on day 5.

Differentiation of the monolayer was assessed by measuring transepithelial electrical resistance (TEER) of the cell monolayers. Only transwell inserts with a resistance exceeding 400 Ω were utilized in the experiments.

### 2.5. Glucose Transport Assay

For glucose transport assay, medium was removed, and differentiated cells were washed twice with HEPES buffer (20 mM HEPES, 137 mM NaCl, 4.7 mM KCl, 1.2 mM MgSO_4_, 1.8 mM CaCl_2_) and placed into a new 12-well plate containing 800 μL of HEPES buffer in the basolateral compartment. 250 μL of donor solution consisting of cell culture medium with 13.5 g/L glucose, 1.0 g/L xylitol and the substance of interest at indicated concentrations was then filled into the apical compartment. Afterwards, 50 μL of samples were collected from the basolateral compartment at various time points and TEER was measured to ensure the integrity of the cell monolayer. The glucose content of the samples was then analyzed using high-performance liquid chromtaography (HPLC) analysis. Finally, 100 μL of donor solution from the apical compartment were used to quantitate the remaining glucose concentration.

### 2.6. Extract Preparation

Fresh *Bellis perennis* plants were collected from a local area and dried at room temperature for 1 week. The dried material was ground in a coffee mill for 30 s. For extract preparation, 3 g of the powder were dissolved in 30 mL EtOH (50%). The solution was treated for 20 min with ultrasound, centrifuged and filtered with a household filter. EtOH was blown off with N_2_ and the residues were once again diluted in water. The samples were stored at −20 °C.

### 2.7. Total Internal Reflection Fluorescence (TIRF) Microscopy

CHO-K1 hIR/GLUT4-myc-GFP cells were grown in 96-well imaging plates (35,000 cells/well; Mobitec, Göttingen, Germany) overnight as previously reported [16,17]. Cell culture medium was aspirated off and, after washing the cells with HBSS (VWR, Vienna, Austria), replaced by HBSS (Thermo Fisher, Waltham, MA, USA) for 3 h. The cells were incubated with insulin or *Bellis perennis* extracts dissolved in KRPH buffer and imaged on an Olympus IX-81 inverted microscope in objective-type total internal reflection (TIR) configuration via an Olympus 60× NA = 1.49 Plan-Apochromat objective as described earlier [27,28]. The 96-well plates were placed on an x–y stage (CMR-STG-MHIX2-motorized table; Märzhäuser, Wetzlar, Germany). Scanning of larger areas was supported by a laser-guided automated focus-hold system (ZDC2). The 488 nm emission of the diode laser (Toptica Photonics, Munich, Germany) was used to image green fluorescent protein (GFP) fluorescence. After appropriate filtering, the fluorescence signal was recorded using an Orca-R2 CCD camera (Hamamatsu Photonics, Herrsching, Germany).

### 2.8. Hens Egg Test-Chorioallantoic Membrane (HET-CAM)

The HET-CAM test was used as previously reported [22,23]. Briefly, eggs were incubated at 38 °C for 11 days. The eggs were automatically and constantly turned, checked for fertilization via candling, and the air bladder area was marked. The eggshell was lightly pecked with a pointed pair of tweezers in this area and 300 μL of a buffer solution (HBSS or water) containing the putative blood glucose-lowering substance was added. We tested 3 different plant extracts from *Bellis perennis* that were obtained from an extract library (PECKISH) or homemade. HBSS or water was used to dilute the extracts to a final concentration of ~300 mg/L, which was finally applied with a syringe into the air compartment of the egg. The eggs were placed back in the incubator for 1 and 2 h. After incubation, the eggshell above the air bladder was carefully removed and the eggshell membrane was equilibrated with PBS. In the next step, the eggshell membrane was removed and the chorioallantoic membrane was carefully cut with a micro-scissor. A suitable blood vessel was carefully placed on a plastic pH strip, which was patted dry using filter paper before the vessel was cut, and leaking blood was collected. The blood glucose levels were determined via a blood glucose meter (Accu-Check Performa, Roche Diabetes Care GmbH, Mannheim, Germany). For each time point, at least 10 fertilized eggs were used. Each experiment was repeated at least three times.

### 2.9. High-Performance Liquid Chromatography (HPLC) Analysis

Extract analyses were performed by reversed-phase chromatography using a Thermo Scientific Dionex Ultimate 3000 comprised of a LPG-3400SD pump with built-in degasser, a WPS-3000 U(T)SL cooled autosampler, a temperature-controlled column compartment and a FLD-34000RS diode array detector (DAD) equipped with the Chromeleon software as described recently [15]. Analyte separation was performed on an Accucore C18 column (150 mm × 3.0 mm inner diameter, 2.6 µm particle size; Thermo Scientific). The column temperature was set to 40 °C and the injection volume was 1 µL. Ultraviolet (UV) wavelengths were detected at 260 nm. The analytes were separated by gradient elution with mobile phase A containing 0.1% formic acid (FA) in water and mobile phase B containing 0.1% FA in acetonitrile at a flow rate of 0.5 mL/min. The elution gradient starting conditions were 95% A and 5% B. After 5 min of equilibration time, the proportion of B was increased to 20% at 8 min and to 40% at 12 min, followed by 60% B at 15 min and 80% B at 17 min for 3 min. B was reduced to 5% at 20 min until 25 min.

High-resolution mass spectra were obtained using a Thermo Fisher Scientific LTQ Orbitrap XL with an Ion Max API Electrospray Source operated in negative ionization mode with the following parameters: Capillary Temp, 350 °C; Sheath Gas Flow, 45; Aux Gas Flow, 15; Source Voltage, 3.5 kV; Capillary Voltage, −25 V; and Tube Lens, −90 V. Separations were performed using an Accucore C18 column (150 mm × 3.0 mm inner diameter, 2.6 µm particle size; Thermo Scientific with the same conditions described above). Polyphenols were quantified against known standards where available with concentrations in a linear range from 1–1000 mg/L.

Sugar analysis was carried out as previously reported with minor modifications [15]. A Jasco LC-2000 Plus Series system comprised of an analytical pump with external degasser, auto-sampler, temperature-controlled column compartment, a Jasco RI-2031 Plus detector and a UV-Vis detector equipped with Chrompass software (all from Jasco Corporation, Tokyo, Japan) was used. Analysis of glucose and xylitol was conducted using the same HPLC system. Separation was performed on a Varian, Meta Carb 87H (PN A5210, SN 12509907) column. The column temperature was set to 56 °C, and isocratic elution was carried out at 0.8 mL/min. A mobile phase of 5 mM sulfuric acid in ddH_2_O was used. HPLC was calibrated with glucose (ranging from 10 to 1000 mg/L) and xylitol (ranging from 5 to 1000 mg/L). The obtained standard curve was linear within this range. The limit of detection (LOD) was defined as a signal-to-noise ratio of 2:1 and limit of quantitation (LOQ) as 4:1. LOD was 2.5 mg/L and LOQ5 mg/L for glucose and xylitol, respectively. Data were processed by Jasco Chrompass Chromatography System software (version 1.7.403.1).

### 2.10. Data Analysis

Initial imaging recordings were supported by the Olympus Xcellence RT software. In-depth analysis for the calculation of the fluorescence intensity in individual cells and a fast comparison of the fluorescent signal in numerous cells at different time intervals was performed using the Spotty framework. Spotty can be retrieved online at http://bioinformatics.fh-hagenberg.at/projects/microprot/. Statistical analysis was performed using 2-way Anova and unpaired *t*-test in Graphpad Prism (version 6.07). Figures were prepared using Corel Draw (version X6).

## 3. Results

### 3.1. Induction of GLUT4-Translocation by Bellis Perennis Extracts

**Figure 1:**
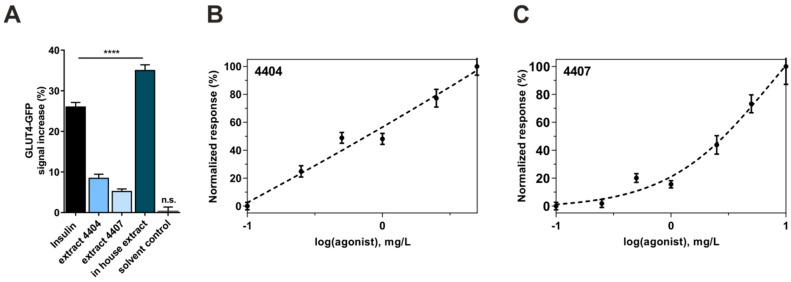
Effects of extracts prepared from *Bellis perennis* on GLUT4 translocation. (**A**) CHO-K1 GLUT4-myc-GFP cells were seeded in 96-well plates (35,000 cells per well), grown overnight followed by 3 h of starvation in Hank’s balanced salt solution (HBSS) buffer, and stimulated by insulin (100 nM) or extracts (1 mg/L) dissolved in Krebs Ringer phosphate HEPES buffer (KRPH) buffer for 10 min. Fluorescence was normalized to the value before insulin application. Error bars are based on the standard error of the mean (*n* > 100, measured on 6 different days). **** *p* < 0.0001. (**B**,**C**) CHO-K1 GLUT4-myc-GFP cells were seeded in 96-well plates, grown overnight and then starved for 3 h in HBSS buffer followed by the addition of various *Bellis perennis* extract concentrations (10 min incubation time). A normalized dose-response curve was generated by measuring the increase in the green fluorescence protein (GFP) signal in the evanescent field after application of the indicated extract concentrations (4404 in (**B**) and 4407 in (**C**)). Error bars are based on the standard error of the mean (SEM) (*n* > 30, measured on 3 different days).

During the last two years, we used a GLUT4-translocation quantitation-based primary screen to identify herbal extracts with insulin mimetic properties [16]. This approach led to the identification of *Bellis perennis* as a potential positive hit. Here, we describe the efficacy of various *Bellis perennis* extracts in inducing the translocation of GLUT4 from cytosolic storage compartments to the plasma membrane. First, two extracts identified from the PECKISH extract library were tested. One extract was a mixture of flowers and leaves (4404) and the other one was produced from flowers alone (4407). Next, we produced an extract prepared from *Bellis perennis* flowers collected from a local area. For the measurements, starved CHO-K1 cells stably expressing the human insulin receptor and a GLUT4-myc-GFP fusion protein were incubated with the chosen extract at low concentrations (1 mg/L). Subsequently, the increase in the GFP signal in the evanescent field, which correlates with the concentration of GLUT4 proteins in the plasma membrane, was determined. Figure 1A indicates the effects of the three tested extracts after 10 min of incubation. Extracts 4404 and 4407 only led to a moderate increase in the GFP signal of ~8% and ~5%, respectively. However, the homemade ethanolic extract resulted in a strong increase in the GFP signal (~35%). To ensure assay performance, we also treated the cells with 100 nM human insulin or the extract solvent (KRPH buffer) only. Insulin led to an increase of ~26%, which is in agreement with previously performed studies [17], while incubation with KRPH buffer did not result in a significant signal increase.

Based on these results, we quantified the efficacy of the two PECKISH library extracts (4404 and 4407) by testing various concentrations ranging from 0.1 mg/L to 10 mg/L. As shown in Figure 1B,C, we found a clear dose response relationship for both extracts, with 4404 being slightly more efficient than 4407. However, there was no effect of 4407 at 0.25 mg/L, while 4404 at the same concentration only resulted in an increase of ~4%. Taken together, the extracts prepared from *Bellis perennis* are effective inducers of GLUT4 translocation in the absence of insulin.

### 3.2. Bellis Perennis Reduces Blood Glucose Levels In Ovo

**Figure 2:**
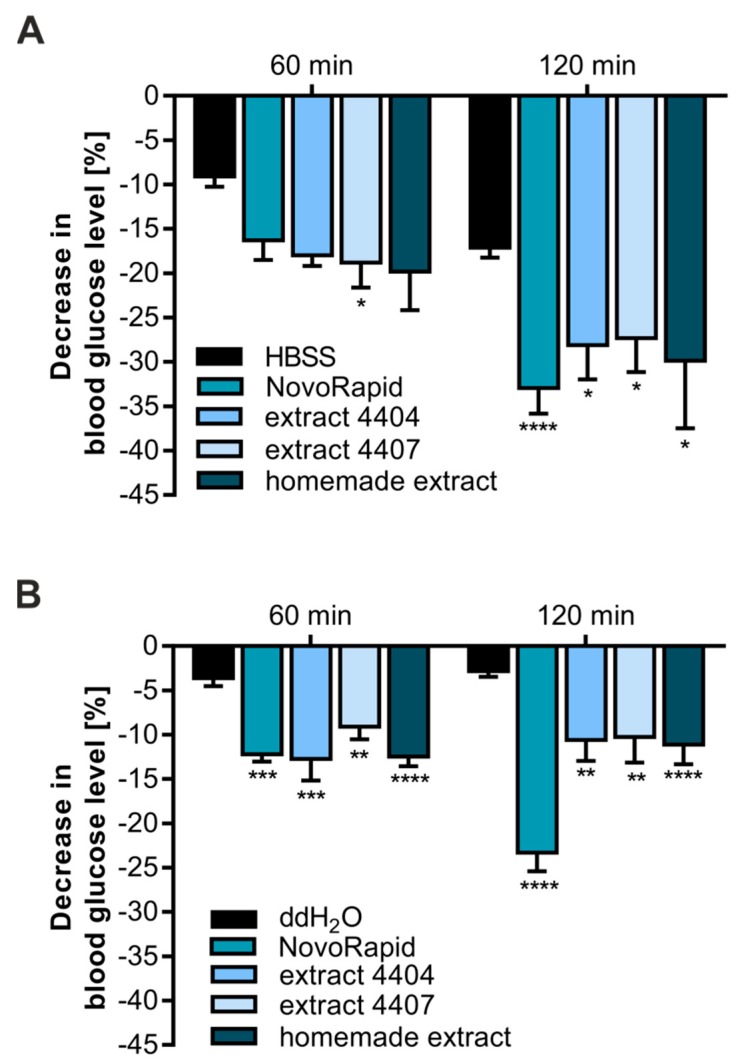
Influence of extracts prepared from *bellis perennis* on blood glucose levels in ovo. Eggs were incubated for 11 days and treated with the indicated substances (NovoRapid: 3.3 U/mL; extracts: 300 mg/L) dissolved in HBSS buffer (**A**) or ddH_2_O (**B**) (300 μL volume) for up to 2 h. Blood glucose levels were determined with a blood glucose meter. Error bars are based on the standard error of the mean. * *p* < 0.05, ** *p* < 0.01, *** *p* < 0.001 and **** *p* < 0.0001, with a significant decrease with respect to HBSS or ddH_2_O treated eggs of the same incubation time.

Based on the results obtained from our CHO-K1-based in vitro system, we decided to test the efficacy of *Bellis perennis* extracts in ovo. For this purpose, we applied our recently established HET-CAM [22,23]. Using this approach, it is possible to test whether the application of a selected compound or extract is effective at reducing blood glucose levels in a living organism. Therefore, the homemade extract as well as both PECKISH extracts were dissolved in HBSS buffer (300 mg/L). NovoRapid, a rapid-acting human insulin analog, was used as a positive control (3.3 U/mL) to prove insulin sensitivity. After incubation with the extracts for 1 and 2 h, blood glucose levels were measured via a blood glucose meter. As shown in Figure 2A, all three extracts resulted in a comparable decrease in blood glucose levels (~20% after 1 h and 30% after 2 h) and were statistically significant after 2 h incubation time. Treatment with NovoRapid led to a reduction of ~16% after 1 h and to a significant effect of ~33% after 2 h.

We have recently shown that HBSS buffer alone also leads to a small decrease in blood glucose levels. Additionally, ddH_2_O has been proven to be a better solvent for assay performance [22]. Therefore, we repeated the experiments described before under these conditions. As demonstrated in Figure 2B, ddH_2_O alone resulted in a non-significant reduction of ~3%, confirming its preferred applicability. Additionally, the three *Bellis perennis* extracts were found to significantly reduce blood glucose levels at both time points with comparable efficacy (~12% after 1 and 2 h).

In conclusion, extracts prepared from *Bellis perennis* are effective at reducing the blood glucose concentration in vitro as well as in a living organism.

### 3.3. Investigation of Putative Negative Effects of Bellis Perennis Extracts on Epithelial Integrity

**Figure 3:**
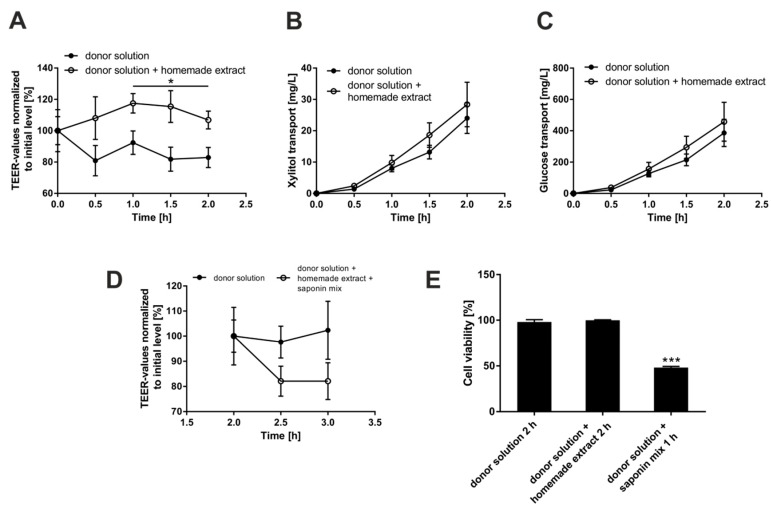
Effects of the home-made *bellis perennis* extract on epithelial membrane integrity. Caco-2 cells were grown on collagen-coated 0.4 µm transwell inserts for monolayer formation and fast differentiation. On day 5, glucose and xylitol transport across the cell monolayer was quantitated. Cell culture medium with 13.5 g/L glucose and 1.0 g/L xylitol was placed as donor solution in the apical compartment. Samples were collected from the basolateral compartment (HEPES buffer) at the respective time points. Glucose and xylitol concentrations of the samples were measured by high-performance liquid chromatography (HPLC). Influence of the extract (**A**) and the extract in combination with a saponin mix (**D**) on the membrane integrity as evaluated by transepithelial electrical resistance (TEER) measurements. Effect of the extract on the cumulative xylitol (**B**) and glucose (**C**) transport from the apical to the basolateral side of Caco-2 monolayers. (**E**) Influence of the used formulations on cell viability. Error bars are based on the standard error of the mean (*n* = 6 inserts, measured on two different days). * *p* < 0.05, *** *p* < 0.001.

Our performed Gluc-HET tests did not lead to observable toxic effects, such as lesions or disordered blood vessels, upon treatment with *Bellis perennis* extracts. To further rule out a reduction of blood glucose levels in ovo due to an unspecific leakage caused by the extracts, we used the Caco-2 monolayer in vitro approach [15]: Caco-2 cells can be grown and differentiated to polarized epithelial cell monolayers on membrane inserts, and the monolayer integrity can be validated by measuring the trans-epithelial electrical resistance (TEER). Incubation with the home-made *Bellis perennis* extract did not lead to reduced TEER values or increased transport of glucose or the sugar alcohol xylitol (Figure 3A–C). Assay performance was validated by addition of a saponin mix, which led to a fast drop of the TEER values (Figure 3D): the saponin mix reduces cell viability (Figure 3E) and thereby increases the para-cellular transport of nutrients including sugars. In conclusion, the *Bellis perennis* extracts apparently do not negatively influence epithelial integrity.

### 3.4. Identification and Quantitation of Polyphenols in Bellis Perennis Extracts

**Figure 4:**
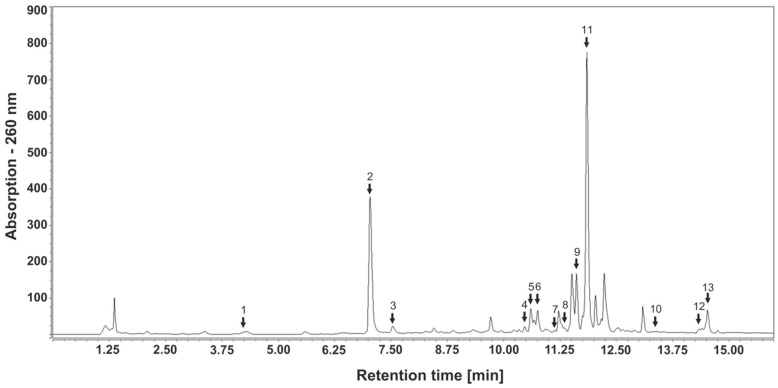
HPLC-diode array detector (DAD) chromatogram of home-made *Bellis perennis* extract recorded at 260 nm. For peak numbers, refer to Table 1.

It is known that several polyphenolic compounds, such as gallic acid, tannic acid, abscisic acid [16,21,29,30], caffeic acid [31] and quercetin [32], are putative anti-diabetic compounds, acting as inducers of GLUT4 translocation. Therefore, we analyzed the polyphenolic contents of the extracts prepared from *Bellis perennis* applied in this study using HPLC-mass spectrometry (MS). First, compounds were identified using mass spectrometry and UV spectra followed by quantitation using calibration curves with the relevant standards. Thirteen polyphenolic compounds were identified and quantified, including rutin, hyperoside, isoquercitrin, guaijaverin, avicularin, quercitrin, quercetin (flavonols), apigenin-7-glucoside (also known as apegetrin), apigenin 7-glucuronide, apigenin (flavones), neochlorogenic acid, chlorogenic acid and caffeic acid (hydroxycinnamic acids). Additionally, kaempferol and luteolin were identified by HPLC-MS analysis, but due to lacking standards and overlapping retention times, they were not quantitated. A representative HPLC-DAD chromatogram indicating retention times and the maximal wavelengths of each compound are shown in Figure 4. Table 1 summarizes the identified polyphenolic contents. The most prominent hydroxycinnamic acid was chlorogenic acid (2.69 mg/L), apigenin-7-glucoside and apigenin-7-glucuronide (overlapping retention times) were found to be highly abundant flavones (0.42 mg/L), and quercitrin was detected as the major flavonol (0.1 mg/L). All other polyphenols were only detected at lower concentrations. Generally, the concentration of polyphenolic compounds in the home-made extract was approximately ten times higher than in the PECKISH extracts. However, the polyphenolic profile obtained from the respective chromatograms was highly similar, indicating identical compositions. In conclusion, polyphenolic compounds are abundant in extracts prepared from *Bellis perennis* and might contribute to the blood glucose reducing effects.

## 4. Discussion

In 2012, diabetes was the eighth leading cause of death among both sexes, leading to 1.5 million deaths worldwide [2,33]. Population growth, the increase in the average age of the population, and especially the rise in diabetes prevalence at each age has steadily increased the number of people living with diabetes [2]. Elevated blood glucose levels, even if below the diagnostic threshold for diabetes (>7.0 mmol/L), represent a key source of morbidity and mortality. Affected people directly suffer from microvascular complications leading to neuropathy or diabetic foot syndrome [34,35], but also macrovascular diseases including an increased risk of heart attack or stroke [36].

As the numbers of people living with diabetes continue to rise, the already-large health and economic impacts of diabetes will also grow. These impacts can be reduced long term through population-based and individual prevention measures that target key risk factors. Hence, there is great demand for pharmaceutical products to treat and, more effectively, prevent diabetes. In this context, phytochemicals potentially serve as an attractive alternative to synthetic medication, as they are associated with a reduced incidence of side effects and low costs. However, low bioavailability and effectiveness might limit their application.

In our lab we are aiming to identify anti-diabetic phytochemicals based on two strategies. First, herbal compounds and extracts that inhibit intestinal glucose resorption using Caco-2 monolayers as an in vitro model have been identified [37,38]. This approach led to the characterization of various positive hits such as extracts prepared from guava fruits and leaves [15]. Second, using a fluorescence microscopy-based in vitro assay, we are screening for plant extracts that induce the translocation of GLUT4 into the plasma membrane [16]. GLUT4 is the main insulin-sensitive glucose transporter in muscle and adipose tissue. Its effective translocation from cytosolic storage compartments to the plasma membrane results in a fast decrease of blood glucose levels [39]. GLUT4 trafficking is frequently disrupted in T2DM, leading to a pathological condition termed insulin resistance (IR) [40]: In this case, cells fail to respond normally to insulin, which finally results in hyperglycemia.

Application of insulin-mimetic compounds, i.e., substances that induce GLUT4 translocation in the absence of insulin, represents a promising strategy for the prevention and treatment of T2DM. Various phytochemicals have been reported to induce the translocation of GLUT4 in vitro [20,41]. Using the aforementioned fluorescence microscopy approach, we confirmed the efficacy of known herbal extracts in inducing GLUT4 translocation [17]. Additionally, we screened hundreds of water-soluble plant extracts from the PECKISH library [26], which resulted in several positive hits. For the screening procedure, the application of the extracts was conducted at a low concentration (1 mg/L) to ensure identification of only effective extracts. We were able to identify two extracts (4404 and 4407) prepared from *Bellis perennis* (common daisy) as potent compound mixtures capable of inducing GLUT4 translocation. Concentrations of only 250–500 µg/L, which are 100–1000 times lower than the one reported for various others, mainly ethanolic/methanolic, plant extracts [42,43,44,45,46], resulted in a significant increase of GLUT4 molecules in the plasma membrane. Additionally, the homemade ethanolic *Bellis perennis* extract prepared from raw material collected from the local area proved to be even more potent in inducing GLUT4 translocation. Application of 1 mg/L resulted in an increase of more than 35%, which was comparable to the effect of the insulin control. We conclude that water-soluble as well as ethanolic *Bellis perennis* extracts are effective inducers of GLUT4 translocation in vitro. However, based on previous studies, we know that a strong increase of the GFP signal in the evanescent field does not necessarily prove an increased membrane insertion: a polyphenolic compound mixture, PP60, was found to increase GLUT4 translocation, but not plasma membrane insertion [17]. Thus, further experiments are required to address this question.

For this purpose, we used a modified hens egg test developed in our lab (termed Gluc-HET; [22,23]) to confirm the efficacy of *Bellis perennis* extracts in reducing blood glucose levels in a living organism (in ovo). In comparison to in vitro assays, in ovo tests are much more time-consuming. Therefore, only compounds with high efficacy in cell culture systems are analyzed. In the course of this study, several plant extracts that led to an induction of GLUT4 translocation in vitro were tested using the Gluc-HET approach. Interestingly, only *Bellis perennis* extracts were capable of significantly reducing the blood glucose concentration of the chicken embryos. This effect was apparent for all three tested extracts in different solvents and at different time points. Interestingly, we did not find significant differences between the water-soluble and homemade *Bellis perennis* extracts in ovo. This was surprising to us, as the concentration of polyphenolic compounds was considerably higher in the home-made extract, in comparison to the PECKISH extracts. We speculate that the available polyphenolics in the PECKISH extracts are sufficient to induce the observed effects and that a higher concentration does not increase their efficacy. Furthermore, we cannot exclude that unknown, non-polyphenolic bioactive compounds are, eventually partly, responsible for the physiological effects. However, we conclude that application of extracts prepared from *Bellis perennis* reduce blood glucose levels in a living organism.

Using Caco-2 cells, which form epithelial like cell monolayers in vitro, we could show that the home-made *Bellis perennis* extract does not reduce the epithelial membrane integrity. This is a good hint that the observed reduction of blood glucose levels in ovo is really caused by an increased uptake into muscle and adipose tissue, rather than an unspecific leakage from the embryonic blood vessels. The Gluc-HET system does have several advantages in comparison to other in vivo models: it is fast, reliable, cheap and no permission by an ethics committee is required. In addition, the chicken embryos are sensitive to human insulin during selected development stages, even though production of insulin has not started. However, we are aware that further in vivo (diabetic mice) and clinical data are required to prove the efficacy of *Bellis perennis* extracts in humans.

We used HPLC-MS for the identification and quantitation of potential active compounds. As polyphenolics are frequently associated with potential anti-diabetic effects in conjunction with elevated GLUT4 translocation [47,48,49], our main focus was on phytochemicals. To date, there is only limited information on the polyphenolic contents of *Bellis perennis*. Identified compounds present in extracts include flavonoids, anthocyanins, tannins and some phenolic acids [50,51,52,53,54]. Our measurements led to identification and quantitation of 13 polyphenolic compounds including 7 flavonols, 3 flavones and 3 hydroxycinnamic acids. Apigenin-7-*O*-glucoside (apigetrin) and apigenin-7-*O*-glucuronide were found to be the most abundant flavones (with overlapping retention times), though aglycon apigenin was only present in very low concentrations. The most prominent hydroxycinnamic acid was chlorogenic acid, while quercitrin was detected as the major flavonol. We could also identify kaempferol and luteolin as two further flavonols. However, due to their identical molecular masses, similar retention times in the UV spectrum and the lack of appropriate standards, these compounds could not be quantitated. In comparison to other studies [51,53,54], we did not find ferulic and rosmarinic acid, isorhamnetin or myricetin. Importantly, PECKISH extracts 4404 and 4407 and the homemade extract resulted in similar chromatograms with only slightly varying peak sizes, indicating identical polyphenolic compound patterns in all extracts used for this study.

When examining the literature, we realized that the putative anti-diabetic effect of *Bellis perennis* extracts described in this study is novel. *Bellis perennis* is known as a traditional herb that has been applied for the treatment of wounds and broken bones for many years [55]. Moreover, it has been used to treat sore throat, headache, common cold, gastritis, enteritis, inflammation and several other diseases in traditional medicine [56,57,58]. Recent studies have indicated that extracts prepared from *Bellis perennis* inhibit the increase of triglyceride levels, showed pancreatic lipase inhibitory activity, induced gastric emptying in olive oil-loaded mice, and promoted collagen synthesis activity in normal human dermal fibroblasts [59,60,61,62]. However, the insulin mimetic and potential anti-diabetic property of extracts prepared from *Bellis perennis* has not been reported so far. We speculate that the particular polyphenolic content might contribute to the effects described here, as it is also the case for other plant extracts, such as extracts prepared from *Portulaca oleracea* [17]. However, we cannot exclude that other bioactive substances are also of relevance. For example, *Bellis perennis* is rich in saponins including bayogenin [63]. These amphipathic compounds also come under consideration, potentially together with polyphenolics, for the documented anti-diabetic effect.

In conclusion, we could show that extracts prepared from *Bellis perennis* are rich in polyphenolic substances, induce GLUT4 translocation in vitro at low concentrations, and effectively reduce blood glucose in live animals. Based on our results, an application in specialized functional food products and food supplements appears reasonable.

## Figures and Tables

**Table 1:** Identification of phenolic compounds in *Bellis perennis* extracts using HPLC with DAD and Orbitrap MS. n.q., not quantifiable; l.o.d., below limit of detection.

Peak	Retention Time, t_R_ [min]	Compound	Mass Spectrometry	Concentration	Concentration	Concentration
Number			(M-H)-	[mg/mL]	[mg/mL]	[mg/mL]
			[*m*/*z*]	Home-Made Extract	Extract 4404	Extract 4407
		**Hydroxycinnamic acids**				
1	4.22	Neochlorogenic acid	353.087	0.0261	0.0053	0.0094
2	7.04	Chlorogenic acid	353.087	1.6904	0.1605	0.2280
3	7.50	Caffeic acid	179.0352	0.0302	0.0059	0.0090
		**Flavonols**				
4	10.46	Rutin	609.1454	0.021	0.0040	0.0026
5	10.6	Hyperoside	463.088	0.0431	0.0020	0.0035
6	10.68	Isoquercitrin	464.0961	0.0567	0.0016	0.0111
7	11.22	Guaijaverin	433.0776	0.0456	0.0023	0.0048
8	11.4	Avicularin	433.0774	0.0115	0.0011	0.0038
9	11.67	Quercitrin	447.0933	0.1036	0.0297	0.0170
10	13.5	Quercetin	302.0433	0.0022	0.0002	l.o.d.
13	14.59 and 14.68	Kaempferol and Luteolin	285.0403	n.q.	n.q.	n.q.
		**Flavones**				
11	11.96 and 12.00	Apigenin-7-glucoside and Apigenin-7-glucuronide	431.0982	0.423	n.q.	n.q.
12	14.34	Apigenin	269.0444	0.0055	0.0001	0.0040
